# Livestock species as emerging models for genomic imprinting

**DOI:** 10.3389/fcell.2024.1348036

**Published:** 2024-02-15

**Authors:** Jean-Noël Hubert, Mathilde Perret, Juliette Riquet, Julie Demars

**Affiliations:** GenPhySE, Université de Toulouse, INRAE, ENVT, Castanet Tolosan, France

**Keywords:** parent-of-origin methylation, large offspring syndrome, callipyge, mammary gland, feeding behaviours, birthweight, epigenome evolution, pathophysiological models

## Abstract

Genomic imprinting is an epigenetically-regulated process of central importance in mammalian development and evolution. It involves multiple levels of regulation, with spatio-temporal heterogeneity, leading to the context-dependent and parent-of-origin specific expression of a small fraction of the genome. Genomic imprinting studies have therefore been essential to increase basic knowledge in functional genomics, evolution biology and developmental biology, as well as with regard to potential clinical and agrigenomic perspectives. Here we offer an overview on the contribution of livestock research, which features attractive resources in several respects, for better understanding genomic imprinting and its functional impacts. Given the related broad implications and complexity, we promote the use of such resources for studying genomic imprinting in a holistic and integrative view. We hope this mini-review will draw attention to the relevance of livestock genomic imprinting studies and stimulate research in this area.

## 1 Introduction

Controlled experiments in mice and investigation of placental pathologies in humans have made it possible to understand the need for a genome derived from specific male and female parental contributions, which must be spatio-temporally regulated for normal development (Hall, 1990; Reik and Walter, 2001). Such a conception of heredity, apparently contravening Mendel’s laws, has fueled the emergence of a specific field of research dedicated to characterizing the underlying phenomenon, known as genomic imprinting, and identifying the molecular mechanisms behind the non-equivalence of parental genomes (Ferguson-Smith and Bourchis, 2018; Tucci et al., 2019).

Our understanding of how regions affected by imprinting have evolved and function has expanded considerably (Llères et al., 2021; Kaneko-Ishino and Ishino, 2022; Richard Albert et al., 2023) and we today have increasingly sophisticated resources and tools (Hubert and Demars, 2022; Jima et al., 2022; Akbari et al., 2023) to identify and accurately report the links between nuclear architecture, Imprinting Control Regions (ICRs), imprinted genes, other genes including different RNA species and, finally, phenotypes. While cytosine methylation is key to almost all regulations that affect it, including transgenerational maintenance, imprinting involves multi-scale mechanisms (Monk et al., 2019), giving grounds for multi-omics approaches (Hubert and Demars, 2022). A diversity of non-exclusive patterns, such as the recruitment of CCCTC-Binding Factor (CTCF) (Noordermeer and Feil, 2020), the transcriptional interference of long non-coding RNA (lncRNA) (MacDonald and Mann, 2020) or the presence of microRNA (miRNA) clusters (Malnou et al., 2019), have, for example, been highlighted at imprinted loci. In addition, histone marks affect the methylation at ICRs, representing another layer of regulatory complexity (Sanli and Feil, 2015). Interestingly, the possibility of DNA methylation-independent, histone-based imprinting has been identified in rodents and termed as noncanonical imprinting (Raas et al., 2022; Inoue, 2023; Richard Albert et al., 2023). The rapid initial accumulation of knowledge on imprinting has been largely enabled by the mouse model (Swain et al., 1987; Li et al., 1993; Greally et al., 1998). However, livestock species have also led to very illuminating results (Cockett et al., 1996; Feil et al., 1998; Jeon et al., 1999; O'Doherty et al., 2015), due to their presence in many vertebrate clades, with a particular importance in the Cetartiodactyla order that includes ruminants and pigs, and the availability of experimental livestock resources suitable for research on imprinting. We therefore wish to emphasize livestock research on imprinting, as we believe it can provide valuable basic knowledge and models to link multi-level molecular variations to complex phenotypical changes (Magee et al., 2014; Daigneault, 2022).

In the present article, we first summarize some of the key contributions regarding imprinting in livestock species, primarily around the identification of imprinted genes, from a broad and historical perspective. We then discuss in more detail molecular mechanisms linking variability at imprinted domains, including *IGF2–H19*, *CDKN1C–KCNQ1* and *DLK1–GTL2*, to developmental effects on muscle and growth phenotypes, as established in livestock species. We finally review several current topics in livestock research aiding deeper understanding of the role of imprinting in complex traits related to energy supply or intake, with broad significance for mammalian biology and health.

## 2 Livestock species occupy a pivotal place in research on genomic imprinting

Initially uncovered in the 80s through iconic murine pronuclear transplantation studies, the presence of imprinting in mammalian genomes led to new methodological developments to detect the parent-of-origin (PofO)-specific expression of genomic regions and their associated functions. They notably consisted of designing specific genetic constructs (Cattanach, 1986; Lee et al., 1993) then molecular biology approaches (Hatada et al., 1993) in mice, allowing the identification of the first imprinted genes in the early 90s (Igf2, Igf2r and H19). In humans, expression studies using in particular fetal tissues allowed determination of the imprinting status of many candidate genes for imprinting (Zhang and Tycko, 1992; Hatada et al., 1996; Blagitko et al., 2000; Murphy et al., 2001) leading to the identification of dozens of imprinted genes but also evidence for differences compared to mice (Kalscheuer et al., 1993; Monk et al., 2006).

**FIGURE 1:**
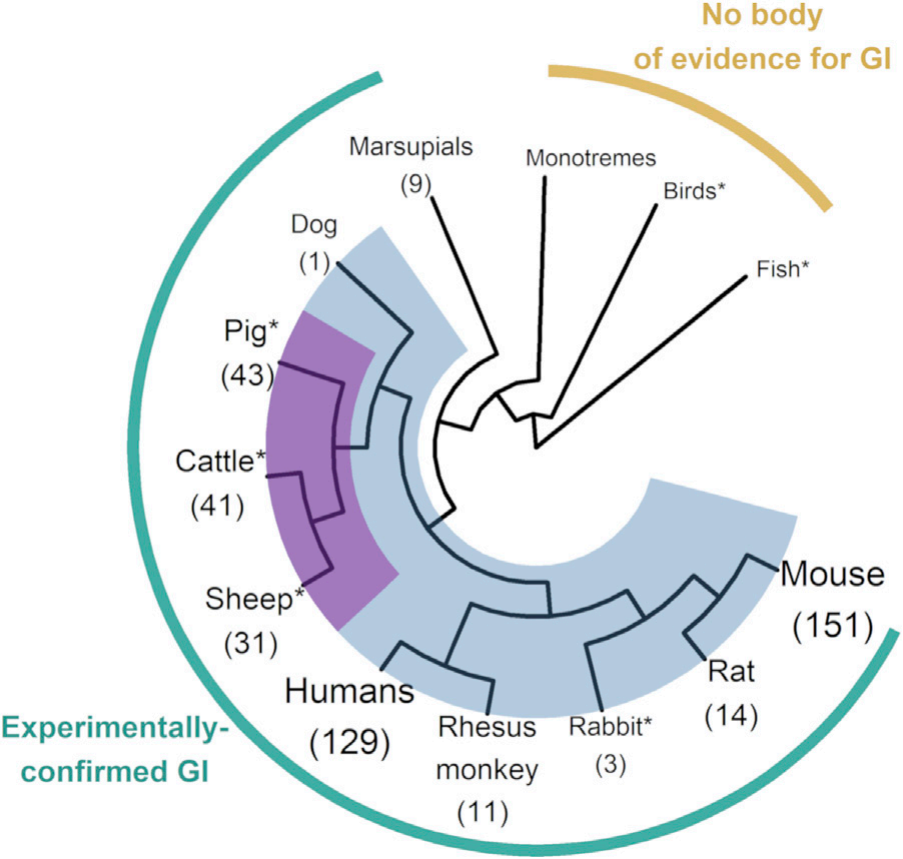
Phylogenetic tree of vertebrates with genomic imprinting research. The figure shows the clades in which the existence of imprinting has been tested. Where imprinted genes have been identified, their total number is reported between brackets under the corresponding clade or species name. Placental mammals, which gather almost all the imprinted genes identified to date, are highlighted in blue. The Cetartiodactyla superorder (including pig, cattle and sheep) is highlighted in magenta, since it forms one of the most documented clades for imprinting, with three branches above 30 imprinted genes identified from data available in livestock species. Size of branch labels is function of the number of currently known imprinted genes in the corresponding clade or species. This figure was produced using R base (v4.1.1) with the ggplot2 (v3.4.2), rotl (v3.0.14), magrittr (v2.0.3) and ggtree (v3.2.1) packages (https://cran.r-project.org/). Count data for imprinted genes come from the Geneimprint Database (http://www.geneimprint.com/). *Clade including livestock species. GI: Genomic imprinting.

In order to check theoretical predictions about the link between imprinting and viviparity, a subset of genes systematically identified as imprinted in placental mammals was found to be imprinted as well in different marsupial species (O'Neill et al., 2000; Suzuki et al., 2005), but not in monotremes (Killian et al., 2001) or in birds (O'Neill et al., 2000). While shedding light on the ancestral origin of genomic imprinting, such single-gene isolation studies were limited to highly conserved imprinted genes, such as in the *IGF2* (Insulin Growth Factor 2) pathway. In 2002, humans and mice became the only mammals with a reference sequence, allowing early genome-wide studies for novel imprinted genes (Luedi et al., 2005; Luedi et al., 2007), in agreement with estimates suggesting that from one to a few percent of all mammalian genes could be subjected to imprinting. Some livestock species soon experienced comparable developments in sequencing technologies, improving the phylogenetic-scale knowledge on imprinting. With pig, cattle and sheep displaying more than 30 experimentally-validated imprinted genes, Cetartiodactyla is one of the most documented mammalian orders on imprinting, alongside primates (Cheong et al., 2015; Chu et al., 2021; Jima et al., 2022) and rodents (Raas et al., 2022; Richard Albert et al., 2023) (Figure 1). Not only do livestock species include representatives from various mammalian lineages but also from key outgroups, i.e., birds and fish, providing opportunities for testing hypotheses on imprinting and epigenomic evolution in vertebrates.

Mainly driven by the needs of different agri-food sectors and the acceleration in the development of sequencing technologies in the 2000s, specific genomic resources began to emerge in animal species of economic importance. In 2015, the NCBI Reference Sequence Database reported at least a chromosome-level assembly reference sequence for a total of 14 of such species, with chicken (Wallis et al., 2004), ruminants (The Bovine Genome Sequencing and Analysis Consortium et al., 2009; The International Sheep Genomics Consortium et al., 2010; Dong et al., 2013) and pigs (Humphray et al., 2007) among the first. At the same time, these species have been subjected to the development of increasingly dense microarray platforms (Sellner et al., 2007). These early annotated genomes and high-throughput genotyping tools have promoted the use of genetic information for breeding, establishing species-specific genetic evaluation procedures (Hayes et al., 2009; Legarra et al., 2011; Ibáñez-Escriche et al., 2014) and enabling functional genomics approaches for increasingly fine and complex traits (Hayes et al., 2010; Do et al., 2013). This also brought higher-resolution analyses of imprinting (Bischoff et al., 2009; Chen et al., 2016; Kenny et al., 2022), leading to the identification of dozens of imprinted genes in pigs, cattle and sheep, including large conserved imprinted domains affecting early development, such as *IGF2–H19* and *MEST* (Rutkowska and Lukaszewicz, 2019).

Combined with valuable pre-existing livestock resources, the increasing availability of Next-Generation Sequencing (NGS) platforms has resulted in diversified studies expanding several areas of our understanding of imprinting. Especially, results from non-mammalian livestock species with both adapted genomic and experimental resources made it possible to raise the question of the presence or absence of imprinting in egg-laying vertebrates (Frésard et al., 2014; Fan et al., 2016) at the genome level. As exemplified in chicken, several genome-scale studies across different tissues and developmental stages are advisable to better understand PofO-specific expression mechanisms among all possible forms of allelic imbalance (Zhuo et al., 2017). Robust tools and procedures are indeed essential when investigating potential imprinting patterns (Edwards et al., 2023), and transcriptomic studies involving reciprocal crosses have so far collectively concluded there were allele-specific expression rather than imprinting in chicken tissues (Frésard et al., 2014; Zhuo et al., 2017). In livestock mammals, NGS-based studies allowed identifying additional imprinted genes and started addressing different related regulatory processes (Congras et al., 2014; Chen et al., 2016; Duan et al., 2018). In addition, a growing body of research explores local mechanisms involved at imprinted loci with evo-devo or biomedical significance (Yu et al., 2018; Ahn et al., 2020; Li J. et al., 2022; Ahn et al., 2023).

Following the path taken by the biomedical research community, international initiatives promote the generation of increasingly comprehensive and accessible tools and resources in epigenomics and transcriptomics (Giuffra et al., 2019; Tixier-Boichard et al., 2021). Recent large-scale cattle studies show the interest of multi-omics not limited to coding regions to capture large proportions of heritability and better predict the phenome (Xiang et al., 2019; Xiang et al., 2023). Efforts in establishing large catalogs of functional elements has resulted in the public availability of datasets from adult, fetal tissues and single cells, including RNAseq, methylation, histone mark, CTCF occupancy and chromatin accessibility data in pigs, cattle and chicken (Kern et al., 2021; Pan et al., 2023). All this novel information on regulatory elements from a diversity of biological contexts (i.e., species, populations, developmental stages, tissues) provides an integrative framework that is particularly helpful to supporting new livestock studies on imprinting (Ahn et al., 2022; Bruscadin et al., 2022).

## 3 Livestock studies reveal a variety of molecular mechanisms at play in genomic imprinting

In livestock species, the major role played by imprinted genes is documented as they are part of the molecular architecture of some agronomic traits (O'Doherty et al., 2015). In pigs and sheep, two mutations affecting a production trait have been identified, and–along with a syndrome linked to imprinting disturbances in cattle–are examples of imprinting contribution to phenotypic variability.

### 3.1 The paternal mutation within *IGF2* is responsible of hypermuscularity in pigs–a regulatory mutation unaffecting methylation

A Quantitative Trait Locus (QTL) with a major effect on muscle mass and fat deposition in pigs has been identified on chromosome 2 from different experimental crosses (Jeon et al., 1999; Nezer et al., 1999). This QTL is responsible for 30% of the variance observed for lean meat, 15%–30% of the variance for muscle mass and 10%–20% of the variance for increased backfat content. The conserved synteny between the porcine region of chromosome 2 and the human orthologous region suggested the paternally expressed *IGF2* gene as a candidate gene given its major role in fetal and post-natal growth in humans. In order to test this hypothesis, an adapted statistical model testing for the presence of an imprinting effect has been developed. A paternal effect of this QTL has been demonstrated, reinforcing the interest of *IGF2*.

The causal mutation of this QTL corresponds to a point mutation in the third intron of the *IGF2* gene (Van Laere et al., 2003). Only pigs homozygous for the mutated allele or heterozygous for the mutated allele inherited from their father show hypermuscularity. Animals carrying the mutated allele on their paternal chromosome produce three times more IGF2 messengers in skeletal muscle after birth (Van Laere et al., 2003). Although the mutation is located in an evolutionarily conserved CpG island that is hypomethylated in skeletal muscle, it does not affect its methylation pattern. However, the mutation abrogates binding to the transcriptional repressor ZBED6 (Zinc finger BED domain-containing protein 6), resulting in an increased expression of messengers from the *IGF2* gene and hence muscle hypertrophy in animals that have received the mutated allele from their father (Markljung et al., 2009).

### 3.2 The *DLK1-MEG3* mutation and the callipyge phenotype in sheep–a unique model of polar overdominance

The Callipyge phenotype, named after a Greek word meaning “beautiful buttocks”, is characterized in sheep by a 30% increase in hindquarters muscle associated with a 8% decrease in fat content and improved feed efficiency (Cockett et al., 1996). The hypermuscularity phenotype is only observed in heterozygous individuals carrying the mutated allele on their paternal chromosome. This atypical mode of non-Mendelian transmission represents a particular case of imprinting known as polar overdominance (Reik and Walter, 2001; Lawson et al., 2013).

The mutation is a substitution located on ovine chromosome 18 within a group of imprinted genes, between the paternally expressed gene coding for the DLK1 (Delta Like Non-Canonical Notch Ligand 1) protein and the maternally expressed *MEG3* (Maternally Expressed 3) gene, which is a lncRNA (Freking et al., 2002; Smit et al., 2003). Callipyge animals, carrying the allele on the paternal chromosome, showed in comparison with non-Callipyge individuals *i)* an overexpression of transcripts from the *DLK1* and *PEG11* (Paternally Expressed 11) genes encoding proteins in skeletal muscle (Murphy et al., 2006), *ii)* a reduction in methylation in muscle throughout the region of the imprinted gene cluster (Takeda et al., 2006), and *iii)* a decrease in the expression of HDAC9 (Histone Deacetylase 9), an enzyme associated with chromatin condensation (Vuocolo et al., 2007). Although two transgenic mouse models for ectopic expression of *DLK1* (Davis et al., 2004) and *PEG11* (Xu et al., 2015) genes have suggested a synergistic action of the two genes to induce the Callipyge phenotype, it has recently been shown that DLK1 seems the primary effector of muscle hypertrophy (Yu et al., 2018).

### 3.3 The large offspring syndrome–a bovine model of human imprinting disorder

The main phenotype of Large Offspring Syndrome (LOS but also known as AOS, Abnormal Offspring Syndrome) is overgrowth, accompanied in particular with macroglossia, umbilical hernia, and limb and spinal cord anomalies; it can be identified during gestation (Rivera et al., 2022). This syndrome has also been observed in fetuses and newborns in ruminants (Walker et al., 1996; Chen et al., 2013). The first cases have been reported in the 90s following the use of assisted reproduction methods such as nuclear transfer (Willadsen et al., 1991) or *in vitro* embryo production (Behboodi et al., 1995). We now know that it can also be of natural origin, but its incidence increases with the use of assisted reproduction techniques (Rivera et al., 2021). In humans, Beckwith-Wiedemann Syndrome (BWS) has a similar etiology. This syndrome, induced in particular by assisted reproduction methods, may be genetic or epigenetic in origin and results in an alteration of imprinting (Filippi and Mckusick, 1970; Brioude et al., 2018).

Interestingly, it has been shown that LOS and BWS are the consequence of a loss of imprinting affecting orthologous genes between the two species (Mangiavacchi et al., 2021). This dysregulation of imprinting concerns the *IGF2–H19* and *CDKN1C–KCNQ1* domains, with their respective ICRs called ICR1 and ICR2 (Li et al., 2022). These alterations involve changes in DNA methylation of either ICR1 (gain of methylation of ICR1 leading to an increased expression of IGF2, an important growth factor (Mangiavacchi et al., 2021)) or ICR2 (loss of methylation of ICR2, leading to a dysregulation of imprinted genes located in the *CDKN1C–KCNQ1* domain). Recent studies have also demonstrated the role of miRNAs (Li et al., 2019), tRNAs (Goldkamp et al., 2022) and changes in chromatin conformation (Li et al., 2022) in the manifestations of BWS. The phenotypic and molecular similarities between the two syndromes, bovine and human, make LOS an excellent model for studying human imprinting disorders.

## 4 Harnessing livestock resources for a fuller picture of the implications of genomic imprinting

Most of our understanding of the phenotypic consequences of imprinting comes from mouse models and human imprinting disorders (Peters, 2014). Imprinting disorders result in a range of clinical features including aberrant pre- and/or postnatal growth and abnormal feeding behaviours (Carli et al., 2020). Imprinted disorders are a group of congenital disorders with common underlying molecular alterations, including genetic abnormalities as well as aberrant epigenetic landscapes, which target imprinted genes. They show that imprinted genes are key loci involved in traits’ variability through their genetic and epigenetic PofO effects. Besides imprinted disorders, more and more studies pinpoint the role of imprinted genes and their PofO effects in the variability of complex traits (Lawson et al., 2013). In livestock species, some traits of major agronomic interest overlap with phenotypes affected in imprinting disorders such as birthweight, postnatal resources through mammary gland development or feeding behaviours, making livestock resources relevant for a better understanding of the role of imprinting in complex traits.

### 4.1 Birthweight is a trait of major importance in pig production

In pigs, birthweight is a trait with an important social impact given its significant association with stillbirth risk and pre-weaning mortality and a major economic interest since it significantly determines later growth and thus impacts economic outputs. Pig genetic selection for reproduction traits has been focused in the last decades on increasing litter size. However, the significant genetic gain obtained for this trait over 20 years of selection was coupled with a reduction of piglet survival from birth to weaning. Moreover, birthweight is considered as a maternal trait in most pig selective breeding neglecting both the direct and paternal effects in birthweight estimation, whereas in humans a paternal contribution to birthweight has been reported (Magnus et al., 2001). Recent epidemiological studies have confirmed that both maternal and paternal genes do participate to infant birthweight, with a greater influence from the maternal side than from the paternal side (Rice and Thapar, 2010). Moreover, estimates of direct heritability for individual birthweight in piglets are moderate (Banville et al., 2015), as also shown in humans (Momoko et al., 2016). As the birthweight of the offspring strongly depends on the maternal environment and genome, the placenta is the fetal tissue which allows the crosstalk between mother and fetus. Interestingly, it shows a tissue-specific imprinting pattern with many genes that are imprinted only in the placenta (Monk, 2015), which makes it a key tissue to better understand birthweight regulation in livestock. Moreover, the differences in placentation between humans, rodents, and pigs suggest that imprinting could serve in pig placenta an additional function to that of placental development and maternal resource allocation (Chavatte-Palmer and Tarrade, 2016).

**FIGURE 2:**
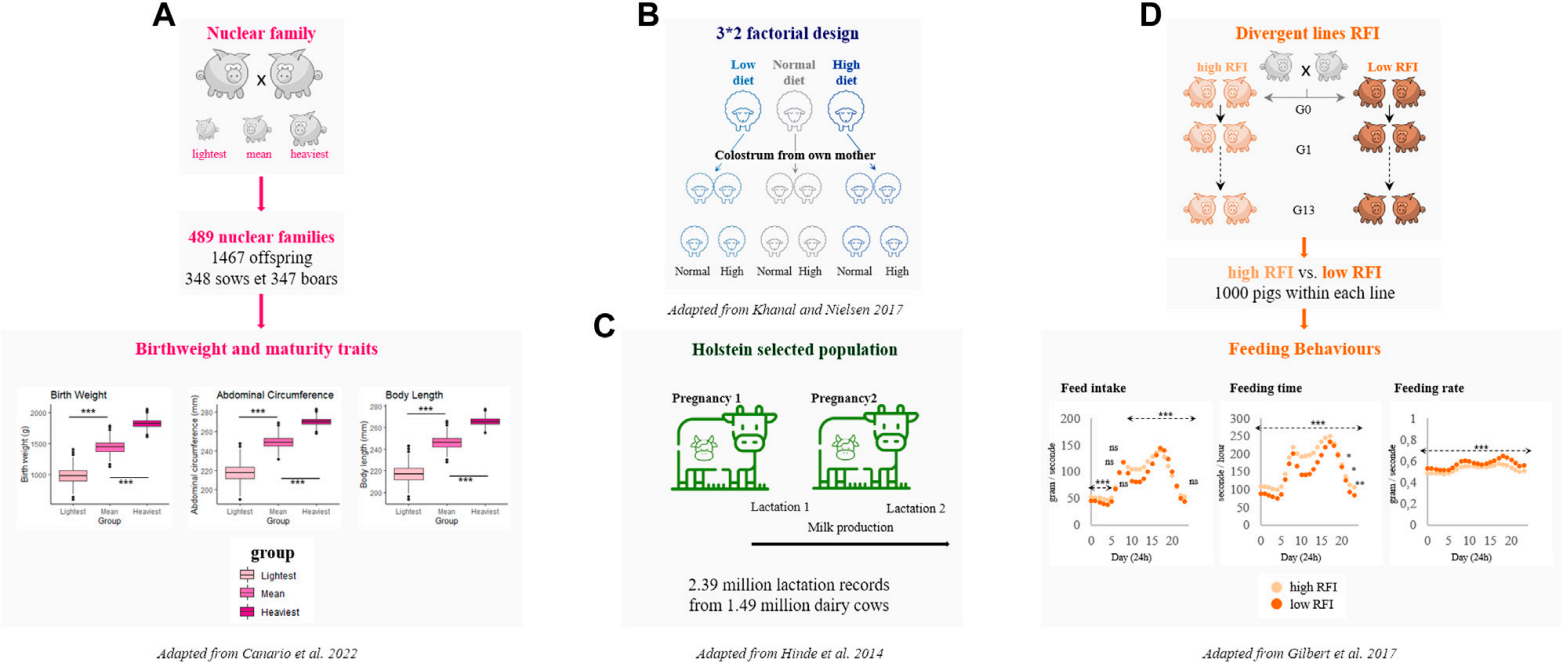
Experimental livestock populations measured for traits overlapping features of imprinted genes. **(A)**- An experimental design has been generated to quantify the different genetic components (paternal, maternal and offspring) of birthweight and maturity in piglets (Canario et al., 2022). **(B)**- The Copenhagen experimental design has been developed in sheep to evaluate impacts of prenatal and postnatal nutrition on animal production and performance (Khanal et al., 2017). **(C)**- The dairy Holstein cow population allow access to over 1 million of lactation records with their associated pedigree (Hinde et al., 2014). **(D)**- Divergent selected lines for Residual Feed Intake (RFI) in pigs over several generations have been also measured for feeding behaviours (Gilbert et al., 2017).

Dedicated experimental designs have been set up to *i)* consider maternal, paternal and direct genetic effects and *ii)* identify genomic regions involved in the molecular architecture of birthweight and more recently maturity of piglets (Canario et al., 2022) (Figure 2A). Many studies have highlighted QTLs affecting birthweight in pigs in different breeds. Most of them are referenced in the Animal QTLdb (Hu et al., 2013). Remarkably, among all QTLs detected for birthweight, several regions encompass clusters of genes that are known to be imprinted in human such as QTLs overlapping with *MEST* (Mesoderm Specific Transcript) and *DLK1* (Guo et al., 2008; Ai et al., 2012). These observations in pigs might mimic results reported in a recent study performed in a familial human dataset that showed an enrichment of associations with birthweight in imprinted regions (Juliusdottir et al., 2021).

### 4.2 The mammary gland–an exocrine organ critical for postnatal growth of the offspring

Although few examples of specific patterns of imprinting in mammary gland have been highlighted (Curley et al., 2004; Andergassen et al., 2017; Xu et al., 2021), its role as a key imprinted tissue is still debated, as for the placenta and the brain. As an example, Grb10 has recently been shown to be maternally-expressed in lactating mammary glands in mice, with complementary functions in mother and offspring. While Grb10 suppresses growth in offspring, it increases milk production in mother (Cowley et al., 2014). Hanin and Ferguson-Smith (2020) support the idea suggested by Stringer et al. (2014) according to which mammary gland represents the functional equivalent of the placenta in the postnatal stage of eutherian mammals, contributing then to the development and growth of offspring. Indeed, mammary gland, by providing milk to the newborn, contributes further to maternal effects on offspring phenotypes, in addition to those that occur during fetal life. Milk provides the neonate with essential nutritional components and non-nutritional bio-active components, such as growth factors including *IGF2* that has been shown to be an important biological regulator of milk production in dairy cattle (Berkowicz et al., 2011). Many studies, mainly in mice, showed that the maternal-offspring interface through lactation is a critical period, suggesting that genetic variation and maternal diets may affect milk composition, leading to lasting effects with alteration of lifelong health (Hanin and Ferguson-Smith, 2020; Rodríguez-González et al., 2020; Lean et al., 2022).

In ruminants, milk production is a major agronomic trait. Failure to produce quality and quantity of milk mainly due to diseases affecting mammary gland leads to high economic losses for breeders. In addition, a report from the Food and Agriculture Organization of the United Nations (FAO) pinpoints the importance of animal milk in the diets of children in populations with very low fat intakes and limited access to other animal source foods (https://www.fao.org/dairy-production-products/products/milk-composition/en/). Association genetics identified many genomic regions influencing different phenotypes of milk composition, showing in particular a major effect of the imprinted gene DGAT1 (Diacylglycerol O-Acyltransferase 1) and caseins on total yields of milk, protein and fat (Poulsen and Larsen, 2021). In addition, other candidate genes mostly affecting milk fat and protein content were shown to play a role in secretory functions in the mammary gland as well as mammary gland development (Cui et al., 2014). In consequence, ruminant species may represent powerful models (Figures 2B, C) to access high throughput information on genetic components of milk composition to fully understand the significance and the physiological role of imprinted genes in the mammary gland and in postnatal provisioning.

### 4.3 Feeding behaviours are indicators of animal welfare and health in livestock

In humans, the known role of imprinting mechanisms in feeding behaviours is mainly restricted to pathologies such as imprinted disorders (Ho-Shing and Dulac, 2019; Elbracht et al., 2020). Typical clinical features of imprinted disorders lies either in difficulties in feeding or hyperphagia. The increasing capacity to record behaviour parameters automatically for livestock precision farming makes it possible to monitor feeding behaviours of individual animals over time (Cellier et al., 2021). Nowadays, investigation of feeding behaviours in livestock species is merely used as a proxy of feed efficiency phenotypes that are major traits in breeding selection schemes (Cavani et al., 2022). For example, feed efficiency represents up to 50% of the breeding objectives in some European pig paternal lines (Gilbert et al., 2017). A divergent selection experiment on a measure of feed efficiency in pigs showed after more than 10 generations an alteration of feeding behaviours traits in feed efficiency-divergent individuals, with significant differences for feed intake (12%), daily eating time (22%) and number of visits and feeding rate (14%) (Gilbert et al., 2017) (Figure 2D).

Only few studies assessing the genomic basis of feeding behaviours and identifying several QTLs have been performed in livestock species so far (Ding et al., 2018; Fu et al., 2020). In an interesting way, among QTLs detected for feeding behaviours and available in Animal QTLdb (Hu et al., 2013), several regions encompass clusters of genes that are known to be imprinted in human such as *DLK1* and *PRKAG2* (Reiner et al., 2009). These observations highlight that livestock might significantly improve the understanding of imprinting in the variability of feeding behaviours, with implications for animal welfare and overall health in sight.

## 5 Discussion

This review aimed to show how livestock species could contribute to essential information on genomic imprinting, from evolutionary conservation to pathophysiological models for specific human disorders, including complex molecular imprinting patterns affecting trait variation.

Overall, there is very little imprinting studies on non-mammalian vertebrates, a first obstacle being the difficulty of accessing or funding adequate experimental resources. The scarcity of such studies may also be partly explained by the widespread possibility of parthenogenesis in these species, which is generally seen as incompatible with imprinting. Non-mammalian vertebrates however show a remarkable diversity of embryo-related adaptations, allowing detailed research on embryonic resource allocation (Furness et al., 2019; Whittington et al., 2022). In both birds and teleost fish, the absence of an orthologue of DNMT3L, a key methylation enzyme in mammals, supports the absence of imprinting. Analyses carried out on whole embryos, adult brains or embryonic tissues always concluded that imprinting was absent in chicken (Frésard et al., 2013). Conversely, studies performed in fish are more contradictory, with Ma et al. showing the *ntl* gene as differentially methylated between sperm and egg in goldfish and a differential timing of transcription between paternal and maternal alleles in the embryo (Ma et al., 2011). Recent data from both laboratory and cultured teleost fish suggest the possibility that some imprinted genes, such as *IGF2* and *DLK1*, present homologues subjected to imprinting-like patterns outside mammals (Du et al., 2022; Zhang et al., 2023), highlighting the need for further research on this topic.

Improved annotation of livestock genomes supported by international initiatives such as FAANG (The FAANG Consortium et al., 2015) and FarmGTEx (https://www.farmgtex.org/) should allow building a more comprehensive phylogenetic tree of imprinting evolution, including investigating potential early differentiation in some clades. As an example, assessment of specific imprinted genes in cattle and pigs showed that SLC38A4 appeared to be species-specific, with a non-imprinted pattern in cattle in all adult tissues evaluated (Zaitoun and Khatib, 2006) compared to mice, which harbor tissue-specific imprinting (Zaitoun and Khatib, 2006). Moreover, a fine-tuned pattern of imprinting has been shown for paternally-expressed NAP1L5, with an expression distribution in pig tissues differing from those in mice and cattle (Jiang et al., 2011). Genome-wide comparative analyses suggest that changes in methylation are a potentially important source for shaping lineage- and species-specific innovations, as exemplified with imprinting (Hu et al., 2023). Livestock mammals such as cattle and pigs display epigenomic-specific features that make them particularly attractive to investigate the contribution of imprinting and epigenomic evolution to phenotypic variation (Lu et al., 2021).

Several traits of agronomic interest overlap with major features of imprinted genes, particularly due to their known role in growth, development and behavioral processes. Although molecular tools targeting imprinting in livestock still need to be developed (Hubert et al., 2024), experimental livestock populations may help to better understand how imprinted genes affect performance (Figure 2). Not only do these populations display a broad range of relevant phenotypes for imprinting (Hinde et al., 2014; Gilbert et al., 2017; Khanal et al., 2017; Canario et al., 2022), but also they allow access to a full pedigree with parental origins either through nuclear family datasets (Khanal et al., 2017; Canario et al., 2022) or divergent lines selected over several generations (Gilbert et al., 2017). Thus, considering parental origins in future genome-wide association studies performed in livestock would enable significant advances on the functional impact of imprinted genes in the variability of important traits.

Apart from the bovine LOS that mimics BWS (Rivera et al., 2022), other livestock species such as pigs might constitute relevant pathophysiological models for imprinted disorders. In mice, targeted mutagenesis has provided highly valuable clues on the mechanisms involved in the regulation of imprinting. However, mouse models and patient data show discrepancies on both growth traits and inheritance patterns (Shmela and Gicquel, 2013), suggesting that mice do not seem appropriate for further analysis of pathophysiology for therapeutic purposes, making pigs a potential alternative model for imprinted disorders. Indeed, pigs are considered as one of the best animal generators of human disease models, because they share similar features with humans in physiology, anatomy and genome organisation (Groenen et al., 2012). The emergence of the CRISPR/Cas9 genome editing technology is revolutionizing porcine genome engineering (Yao et al., 2016). Recent studies have introduced epigenome-editing strategies which target enzymatic activity to introduce or remove an epigenetic mark at a defined genomic site (Kungulovski and Jeltsch, 2016). Given the crucial role of ICRs into the regulation of local imprinted genes and the disruption of DNA methylation observed there in various imprinted disorders (Soellner et al., 2017), epigenome-editing technologies might be applied to perturb DNA methylation at a dedicated ICR in an allele-specific manner to mimic a specific disorder Supplementary Figure S1. Epigenome-edited pigs might be promising pathophysiological animal models to investigate the long-term dynamics of imprinted disorders.

## Supplementary Material

Supplementary Figure 1 Pathophysiological pig model for Silver Russel Syndrome. Silver Russel Syndrome is an imprinted disorder mainly characterized by individuals being born small for gestational age, with relative macrocephaly at birth. The major molecular abnormality of Silver Russel Syndrome is a loss of methylation at the Imprinting Control Center 1 (ICR1) leading to a loss of IGF2 expression from the paternal allele and a biallelic expression of H19. **(A)** Principle of epigenome targeting technology applied to imprinting loci. Schematic representation of a catalytically inactive mutant Cas9 (dCas9) fused with Tet1 for erasing DNA methylation and with Dnmt3a for de novo methylation of specific sequences. **(B)** Schematic representation of the IGF2/H19 imprinting region associated with Beckwith-Wiedemann (excessive growth) and Silver-Russel (growth retardation). **(C)** The polymorphism is exploited to specifically target the paternal allele with the single guide RNA (sgRNA). The system coupled to Tet1 will remove the DNA methylation from the paternal ICR1. This will result to a loss of methylation at ICR1 which is the most common epimutation in Silver-Russell patients. **(D)** Generation of offspring with a loss of methylation at ICR1 that could mimic the Silver-Russel Syndrome. SNP: single nucleotide polymorphism.
