# Supplementary figures and images for: Livestock species as emerging models for genomic imprinting
Source: Front Cell Dev Biol. 2024 Feb 15;12:1348036. doi: 10.3389/fcell.2024.1348036 (PMC10945557; doi:10.3389/fcell.2024.1348036)

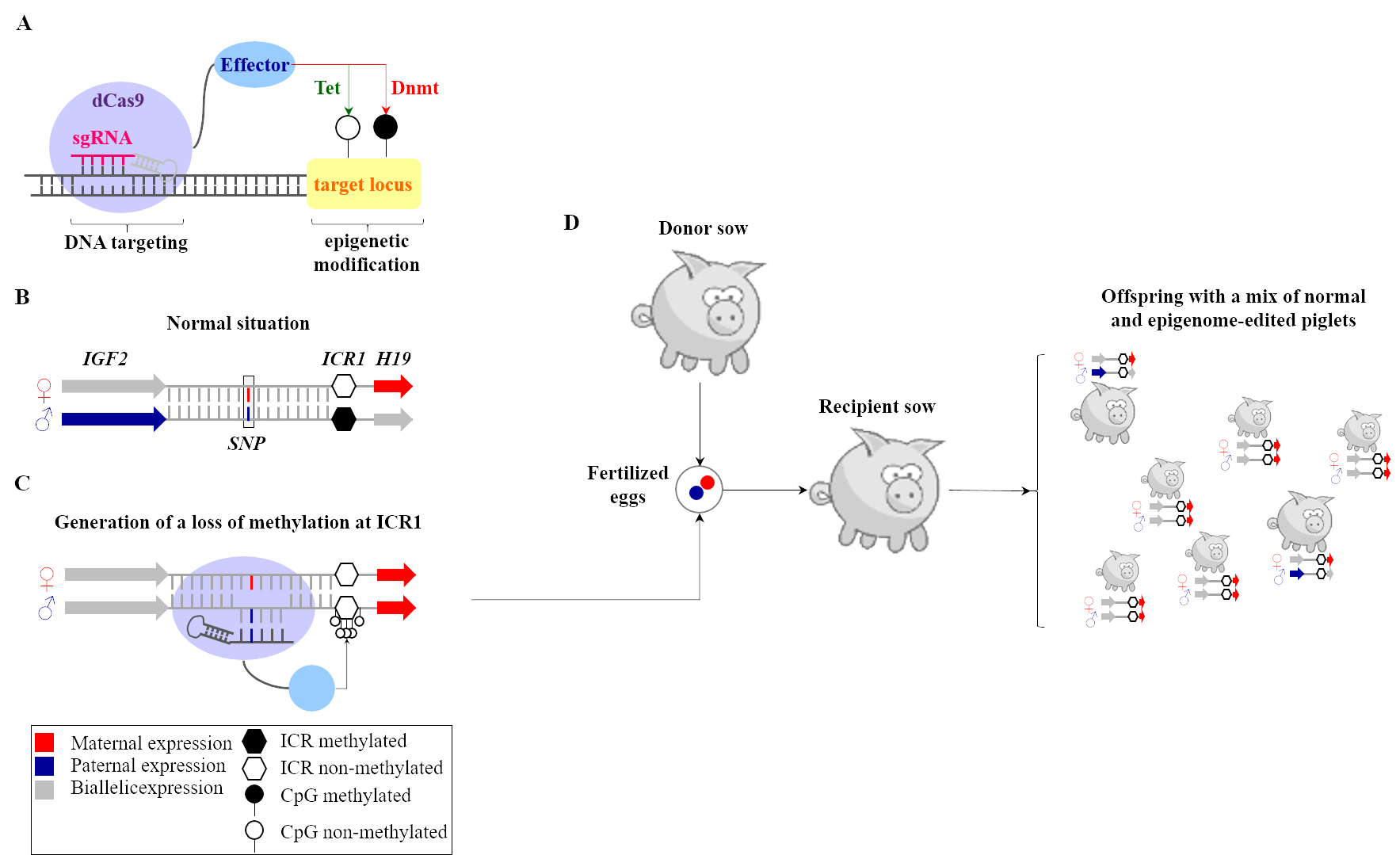

Supplement: Supplementary file 1 [file Image1.tiff]
